# Physicochemical and Rheological Properties of Degraded Konjac Gum by Abalone (*Haliotis discus hannai*) Viscera Enzyme

**DOI:** 10.3390/foods13132158

**Published:** 2024-07-08

**Authors:** Zhao-Ming Lin, Jia-Xin Wen, Duan-Quan Lin, Kang Liu, Yu-Lei Chen, Song Miao, Min-Jie Cao, Le-Chang Sun

**Affiliations:** 1 College of Ocean Food and Biological Engineering, Jimei University, Xiamen 361021, China; zhaoming.lin@jmu.edu.cn (Z.-M.L.); 202312951081@jmu.edu.cn (J.-X.W.); dq.lin@jmu.edu.cn (D.-Q.L.); liukang19880330@jmu.edu.cn (K.L.); ylchen@jmu.edu.cn (Y.-L.C.); mjcao@jmu.edu.cn (M.-J.C.); 2 National & Local Joint Engineering Research Center of Deep Processing Technology for Aquatic Products, Jimei University, Xiamen 361021, China; 3 Collaborative Innovation Center of Seafood Deep Processing, Dalian 116034, China; 4 Teagasc Food Research Centre, Moorepark, Fermoy, P61 C996 Cork, Ireland; song.miao@teagasc.ie

**Keywords:** konjac gum, abalone viscera, enzymatic degradation, rheology properties

## Abstract

In the present study, a new degraded konjac glucomannan (DKGM) was prepared using a crude enzyme from abalone (*Haliotis discus hannai*) viscera, and its physicochemical properties were investigated. After enzymatic hydrolysis, the viscosity of KGM obviously decreased from 15,500 mPa·s to 398 mPa·s. The rheological properties analysis of KGM and DKGMs revealed that they were pseudoplastic fluids, and pseudoplasticity, viscoelasticity, melting temperature, and gelling temperature significantly decreased after enzymatic hydrolysis, especially for KGM-180 and KGM-240. In addition, the molecular weight of KGM decreased from 1.80 × 10^6^ Da, to 0.45 × 10^6^ Da and the polydispersity index increased from 1.17 to 1.83 after 240 min of degradation time. Compared with natural KGM, the smaller particle size distribution of DKGM further suggests enzyme hydrolysis reduces the aggregation of molecular chains with low molecular weight. FT-IR and FESEM analyses showed that the fragmented KMG chain did not affect the structural characteristics of molecular monomers; however, the dense three-dimensional network microstructure formed by intermolecular interaction changed to fragment microstructure after enzyme hydrolysis. These results revealed that the viscosity and rheological properties of KGM could be controlled and effectively changed using crude enzymes from abalone viscera. This work provides theoretical guidance for the promising application of DKGM in the food industry.

## 1. Introduction

Konjac glucomannan (KGM), the main component of konjac gum, is extracted from the root of konjac tuber (Amorphophallus konjac). It is a high molecular weight polysaccharide consisting of D-glucose and D-mannose units via β-1,4-linkage [1,2]. Due to its high thickening, good film formation, excellent gelation, good biocompatibility, and biodegradability, as well as its colorless, odorless, and non-toxic characteristics, KGM is generally regarded as a safe (GRAS) food additive by the FDA and has been widely used in the food industry [3].

However, native KGM displayed extremely high viscosity and poor water dispersibility due to its high molecular weight, which greatly limited its application during food processing [4,5]. Recently, a number of studies have focused on exploring efficient methods for KGM degradation to improve its water solubility, including biodegradation (enzyme hydrolysis) [6,7,8], physicochemical degradations, such as acidic hydrolysis [9], ultrasonic treatment [10], and irradiation degradation [11]. In comparison with physical and chemical treatments, biological treatments, especially enzyme treatments, provide biocompatibility, selectivity, and effectiveness. method for preparing degraded KGM [12]. However, enzyme hydrolysis has high costs, separation difficulties, and specific reactive conditions [13]. Therefore, it is worth exploring a low-cost and high-efficiency enzyme to obtain degraded KGM with low viscosity and high hydrophilicity. Cellulase, including β-mannanase and β-glucanase, is the most commonly used enzyme for KGM degradation due to the breaking of the β-1,4-glycosidic bond between glucose and mannose. Song et al. [8] reported that KGM was effectively degraded by β-mannanase hydrolysis, and the degraded KGM showed a certain inhibition on ACE activity. In addition, the degraded KGM was also produced by β-glucanase to improve the cryoprotective effect on the glass carp myofibrillar during frozen storage [14]. Hence, cellulase shows great potential to efficiently perform KGM degradation.

Abalone is a type of coastal marine herbivorous mollusk, and its production will reach 228,190 tons in China in 2022 [15]. The viscera tissues with a variety of enzymes, such as alginate lyase, β-1,3-glucanase, cellulase, and agarase [16], account for about 20 to 30% of body weight, and they are usually discarded directly or used as animal feed [17]. In our previous study, an endo-1,4-β-glucanase belonging to cellulose was purified from the hepatopancreas of abalone (*Haliotis discus hannai*), showing six-fold specific activity compared to commercial cellulase from *Aspergillus niger* [18]. However, there is lack of information about the enzyme from abalone viscera that degrades KGM. It is reported that the hydration rate and viscosity of KGM are related to its molecular weight, degree of acetylation, particle size, and surface morphology [19].

The objective of the present study is to prepare degraded KGM using the abalone viscera enzyme and evaluate the physicochemical and rheological properties of DKGM. The changes in molecular properties and microstructure of degraded KGM were also investigated to provide a theoretical reference for KGM degradation.

## 2. Materials and Methods

### 2.1. Materials and Reagents

*Haliotis discus hannai* is a widely cultivated edible shellfish in China that is not an endangered or specially protected species. All methods used in this study were conducted in accordance with the guidelines of the Chinese Laboratory Animal Use and Care Legislation.

Live-cultured abalones (*Haliotis discus hannai*) with an average body weight of 80–90 g were purchased from a local aquaculture market in Xiamen, China, and then transported to the laboratory alive in clean oxygenated water. Abalones were exposed and immersed in ice/water slurry (temperature = 1 ± 1 °C) until no prolegs movements were observed. Then, the sacrificed abalone’s hepatopancreas were peeled off carefully under low temperatures to prepare abalone viscera enzyme. Konjac gum with more than 95% konjac glucomannan (KGM) content was obtained from Maillard Food Technology Co., Ltd. (Xiamen, China). All other chemicals were purchased from Sinopharm Chemical Reagent Co., Ltd. (Beijing, China).

### 2.2. Preparation of Abalone Viscera Enzyme

The collected abalone viscera were finely chopped and homogenized with four volumes of 10 mmol/L phosphate buffer (pH 6.0) using a homogenizer (PT-2100, Kinematica, Lucerne, Switzerland). After being centrifuged at 10,000× *g* for 20 min using a centrifuge (Avanti J-26S XP, Beckman Coulter, Brea, CA, USA), the supernatant was pooled and filtered using a 100 kDa membrane (Millipore, Billerica, MA, USA). The collected permeate was regarded as a crude abalone viscera enzyme. All the procedures were performed at 4 °C.

### 2.3. Determination of Abalone Viscera Enzyme Activity

A 50 μL crude enzyme (10 mg/mL, *w*/*v*) was added into 600 μL of 0.5% KGM solution prepared with 10 mmol/L sodium phosphate (pH 6.0). The reaction was performed at 37 °C for 30 min and terminated after being heated at 100 °C for 15 min. Then, the 200 μL of supernatant and 600 μL of 3,5-dinitrosalicylic acid (DNS) were thoroughly mixed and incubated at 95 °C for 15 min to measure the absorbance at 540 nm [2]. The standard curve of released reducing sugar was plotted using glucose as a control. One unit of enzyme activity was defined as the enzyme amount that produces 1 μmol reducing of sugar per min.

### 2.4. Substrate Property of Abalone Viscera Enzyme

Carboxymethyl cellulose sodium (CMC-Na), carrageenan, xanthan gum, KGM, and sodium alginate were used as substrates to determine the substrate property of the abalone viscera enzyme. The relative activity was calculated as the percentage of enzyme activity toward CMC-Na.

### 2.5. Thermal and pH Profiles of Abalone Viscera Enzyme

The temperature profile of the enzyme was measured in 50 mmol/L sodium phosphate (pH 6.0) under different reaction temperatures from 20 to 70 °C, and its pH profile was determined at 37 °C in 50 mmol/L different buffers with pH values from 3.0 to 9.0: glycine-HCl buffer (pH 3.0), acetic acid-sodium acetate buffer (pH 4.0–5.0), phosphate buffer (pH 6.0–7.0), Tris-HCl buffer (pH 8.0), and sodium carbonate-bicarbonate buffer (pH 9.0).

### 2.6. Preparation of Degraded KGM

An abalone viscera crude enzyme (4 U) was added into 100 mL of KGM solution (2%, *w*/*v*) and incubated at 50 °C for 60, 120, 150, 180, and 240 min, respectively. Then, the hydrolysis reactions were terminated after being heated at 100 °C for 15 min. The resultant solutions were collected as degraded KGMs, including KGM-60, KGM-120, KGM-150, KGM-180, and KGM-240, respectively. Undegraded KGM was used as the control group.

### 2.7. Viscosity of Native and Degraded KGMs

The native and degraded KGMs solution (*w*/*v*) with different concentrations from 0–2.5% were prepared, and their viscosity properties were measured by a Brookfield LVDV-E Digital Viscometer (Brookfield Engineering Labs., Inc., Middleboro, MA, USA) at 25 °C.

### 2.8. Molecular Weight of Native and Degraded KGMs

The molecular weight and polydispersity index (PDI) of native and degraded KGMs (5 mg/mL) were measured using a high-performance gel filtration chromatography (HPGFC) instrument (Waters 1525, Waters Co., Milford, MA, USA) with an UltrahydrogelTM Linear column (300 mm × 7.8 mm, Waters Co., USA) and a 2414 refractive index detector at 25 °C. The loading volume, elution speed, and mobile phase were 60 μL, 0.6 mL/min, and sodium nitrate solution (0.1 mol/L), respectively.

### 2.9. Particle Size Distribution of Native and Degraded KGMs

The particle size distribution of native and degraded KGMs (0.01% *w*/*w*) was determined by laser Doppler electrophoresis combined with phase analysis light scattering (Zetasizer Nano ZS, Malvern Instrument, Worcestershire, UK) at 25 °C [20].

### 2.10. Rheological Properties of Native and Degraded KGMs

Rheological tests of native and degraded KGMs (2% *w*/*v*) were conducted by a DHR-2 rheometer (Suwanee, GA, USA) with parallel-plate geometry (40 mm aluminum parallel plate and 1 mm plate gap).

#### 2.10.1. Steady Shear Behavior

The shear rate increased gradually from 0.1 to 100 s^−1^ and then decreased from 100 to 0.1 s^−1^ at 25 °C. Variations in apparent viscosity (η) and shear stress (τ) with shear rate (γ) were recorded [21].

$$\tau =K\gamma^{n}$$

where τ is shear stress (Pa), γ is shear rate (1/s), K is consistency coefficient (Pa·sn), and n is flow behavior index (dimensionless).

#### 2.10.2. Frequency Sweep Measurement

The native and degraded KGMs were subjected to strain in the range of 0.1–100% at a constant frequency (1 Hz) to determine their linear viscoelastic regime (LVR). The strain value of 1% was chosen in other oscillation tests. Then, the frequency sweep test of KGMs with a continuous frequency increase (0.01 to 10 Hz) was carried out at a constant strain (1%) and 25 °C. Storage modulus (G′) and loss modulus (G″) were recorded [22].

#### 2.10.3. Temperature Ramp Test

The native and degraded KGMs were sealed with inorganic oil to prevent evaporation. The prepared samples were heated from 20 to 80 °C and then cooled from 80 to 20 °C at a rate of 2 °C/min. The measurements were carried out at a fixed frequency of 1 Hz with a strain value of 1%. The rheological parameters (G′ and G″) were recorded per 0.5 min [23].

### 2.11. Fourier-Transform Infrared Spectroscopy of Native and Degraded KGMs

The Fourier-transform infrared (FT-IR) spectra of native and degraded KGMs were obtained by infrared spectrophotometers (Vector-22, Bruker, Fällanden, Switzerland) with wavenumbers ranging from 400 to 4000 cm^−1^ and a resolution of 4 cm^−1^ [24].

### 2.12. Field Emission Scanning Electron Microscopy of Native and Degraded KGMs

The native and degraded KGMs solution (2%, *w*/*v*) were mounted on a bronze stub and quickly froze in the frozen machine with a Peltier stage (MK3 Coolstage, Deben UK Ltd., Bury Saint Edmunds, UK). Surface morphological characteristics were observed using field emission scanning electron microscopy (FESEM) (MIRA LMU, TESCAN Ltd., Brno, Czech Republic). The acceleration voltage was 10 kV, and magnifications were set at 1000-fold under high vacuum conditions [24].

### 2.13. Statistical Analysis

All experiments were carried out in triplicate. SPSS version 22.0 and Origin Pro 9.0 were used to conduct data analysis.

## 3. Results

### 3.1. Enzymatic Properties of Abalone Viscera Enzyme

**Figure 1:**
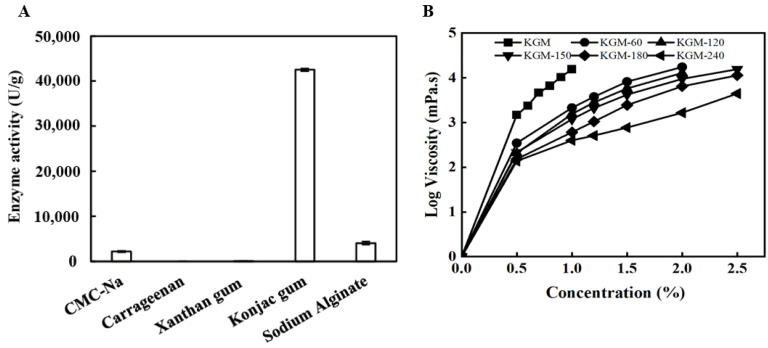
Substrate specificity (**A**) and viscosity-reduction property (**B**) of the abalone viscera enzyme.

The substrate specificity of the abalone viscera enzyme towards five different polysaccharides was investigated, including CMC-Na, carrageenan, xanthan gum, KGM, and sodium alginate. As shown in Figure 1A, it displayed specific activity towards CMC-Na, KGM, and sodium alginate, but little to no significant activity for other polysaccharide substrates. Notably, the specific activity of KGM was 18.6 times greater than that of CMC-Na, indicating that the abalone viscera enzyme has the function to hydrolyze endo-β-1,4-glycosidic bond, according to β-1,4-linked D-glucose and D-mannose residues as the main chains of KGM [20].

Additionally, compared with native KGM, the viscosity of degraded KGM obviously decreased after abalone viscera enzyme treatment, as shown in Figure 1B. When the content of KGM was 1.0%, the viscosity decreased from 15,545 mPa·s to 3800 mPa·s, 1550 mPa·s, 1185 mPa·s, 607 mPa·s, and 398 mPa·s after 60 min, 120 min, 150 min, 180 min, and 240 min, respectively. These results revealed that abalone viscera enzyme hydrolysis is an effective method for KGM degradation, similar to physical treatment such as γ-irradiation [25]. Moreover, the viscosity properties of native and degraded KGMs all presented obvious concentration dependence ranging from 0–2.5% (Figure 1B), further suggesting that the reduction of viscosity was not only attributed to polymer degradation by breakage of the polymer chain or glycosidic linkage but also to the disruption of polymer aggregation [26].

**Figure 2:**
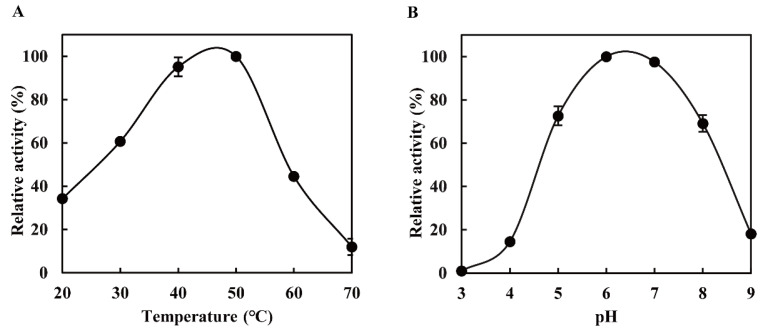
Thermal (**A**) and pH profiles (**B**) of the abalone viscera enzyme.

Using KGM as substrate, the optimal temperature of the abalone viscera enzyme was 40–50 °C (Figure 2A). Its relative activity was still retained at about 60% at 30 °C, suggesting that it can be used to hydrolyze KGM at low temperatures. Moreover, it also exhibited high relative activities in the range of pH 5.0–8.0 (retaining >60% of its maximal activity) (Figure 2B). These results were similar to those of other glucanases from *Paenibacillus polymyxa* [27] and disk abalone from *Haliotis discus hannai*. According to the structural composition of KGM, the KGM degradation enzymes include endo-β-1,4-glucanase (EC 3.2.1.4) and endo-β-1,4-mannanase (EC 3.2.1.78) [27], further confirming that the abalone viscera enzyme belongs to the family of endo-β-1,4-glucanase and has strong tolerance to the environment for its application.

### 3.2. Molecular Properties of Native and Degraded KGMs

Usually, the viscosity and rheological properties of polysaccharides are related to their chain length and molecular flexibility [28]. Hence, the molecular properties of KGMs were further evaluated, and the results are presented in Table 1. The molecular weight of KGM decreased from 1.80 × 10^6^ Da to 1.24 × 10^6^ Da after 120 min, and as degradation processing went on, the molecular weight dropped to 6.93 × 10^5^ Da, 5.71 × 10^5^ Da, and 4.48 × 10^5^ Da for degraded KGM at 150 min, 180 min, and 240 min, respectively. The rapid decline of molecular weight for degraded KGM during 60–240 min is attributed to the disruption of glycosidic bonds within KGM molecular chains after enzymatic degradation, similar to those of KGM treated with ultrasonic degradation [10,29], homogenization pressure [21], and laser irradiation [25].

**Figure 3:**
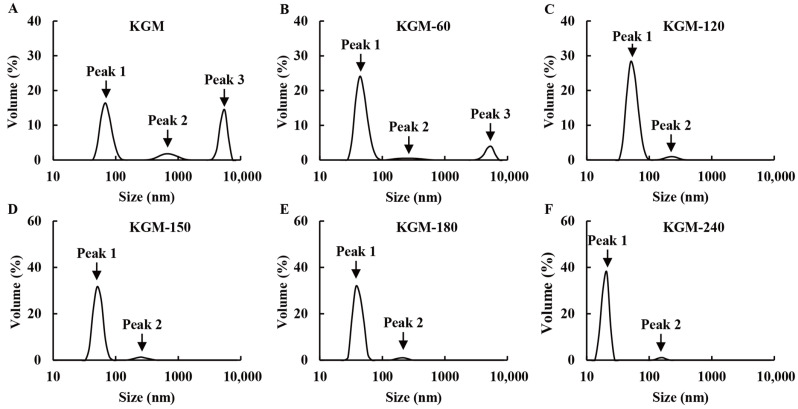
Particle size distribution of native (**A**) and degraded KGMs for 60 min (**B**), 120 min (**C**), 150 min (**D**), 180 min (**E**) and 240 min (**F**) at 25 °C.

In addition, the polydispersity index (PDI) molecular weight/Mn of native KGM was 1.17, while the degraded KGMs for 60 min, 120 min, 150 min, 180 min, and 240 min were 1.22, 1.32, 1.76, 1.88, and 1.83, respectively (Table 1). The slightly increased values indicated that abalone viscera enzyme treatment is a random rupture process [14]. However, all PDI values were less than 2, suggesting that the molecular mass distribution of degraded KGMs was relatively uniform [25,30], which was further proved by the results of particle size distribution. As shown in Figure 3, the native KGM presented three peaks: peak 1 (<300 nm), peak 2 (300–1000 nm), and peak 3 (>1000 nm). The peak 3 corresponding to macromolecular components accounts for 35.1%, which is consistent with the high molecular weight of native KGM (Table 1). After enzymatic degradation, the volume of peak 3 decreased until it disappeared, whereas the area of peak 1 gradually increased during 60–240 min, suggesting the short fractions generated because of the disruption of glycosidic bonds in KGM. These results are in accordance with the reduced molecular weight observed in Table 1. Moreover, the higher volume and smaller size of peak 1 also suggested that these shorter fractions are not associated with large aggregates [11].

### 3.3. Rheological Properties of Native and Degraded KGMs

#### 3.3.1. Steady Shear Behavior

**Figure 4:**
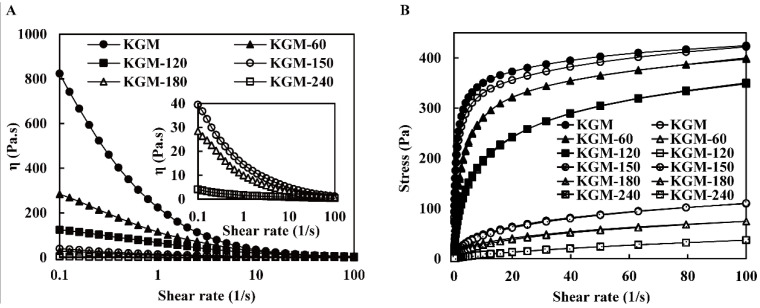
The apparent viscosity (**A**) and flow behavior (**B**) of native and degraded KGMs for 60–240 min. Upward curve: dark; Downward curve: light.

The steady shear behavior of native and degraded KGMs was tested at a shear rate ranging from 0.1–100 s^−1^ (Figure 4A). The flow curves of native and degraded KGMs for 60–180 min decreased with the increasing shear rate, indicating that these KGMs exhibited typical non-Newtonian fluid behavior with shear-thinning behavior. The reduced apparent viscosity of KGMs may be explained by the disruption of the entangled molecular network [31]. At the low shear rate, a new molecular interaction was formed to balance the disruption of the interaction, resulting in a relatively constant apparent viscosity. Whereas, at the high shear rate, the disruption of chain entanglements predominated, and molecules aligned in the direction of flow, leading to a decrease in apparent viscosity. Moreover, the decline of molecular weight and molecular chain length involved in KGM molecules alignment seemed connected with the steady shear behavior [24,30]. The degraded KGMs displayed a continuously reduced apparent viscosity after enzymatic degradation from 60 min to 180 min. Until 240 min, the viscosity of KGM-240 was very low, close to that of water, and turned to a near-Newtonian fluid, as shown in Figure 4A. Commonly, KGM with higher molecular weight tends to establish more entanglements through hydrogen, electrostatic, and hydrophobic bonds [13]. Thus, the shorter molecular chains formed fewer entanglements among inter- and intra-molecular hydrogen bonds [29]. A similar behavior was also observed in other degraded carbohydrates, such as carrageenan, starch, and guar gum. Throughout the entire rheological experiment, KGM and DKGM maintained a semi-solid flow state and did not undergo crystallization.

The native KGM exhibited a distinct hysteresis loop, which is associated with the first round of shear and formed during the second round of shear. Due to the destructive structure, it took some time to recover [32]. However, the ring area of degraded KGMs gradually vanished, especially after 120 min or more (Figure 4B), indicating that the orderly structure formed by short chains for degraded KGMs is more likely to be restored. These results revealed the new structures of degraded KGMs varied from the semiflexible straight chain in the elastic microsphere of native KGM [33], which altered their hydration property, resulting in low viscosity [34].

The power law was widely used to evaluate the dependence of steady shear viscosity on shear rate. Index K and n are the consistency coefficient and flow behavior, respectively. As presented in Table 2, the correlation coefficient (R^2^) ranged from 0.910 to 0.999, suggesting the power law model was well-fitted to investigate the rheological property of KGM. All index n were lower than 1.0, revealing that all KGMs had pseudoplastic manners [29]. With the increase in enzymatic treatment time, index n increased and index K decreased (Table 2). The results suggested that the degraded KGM presented more pseudoplastic properties and better fluidity, which is in accordance with the change in KGM treated with increasing irradiation doses [11]. Usually, a molecular chain with a higher molecular weight entanglement density results in a longer relaxation time and a higher K value [35]. Thus, the low K values revealed that the degradation of KGM affects its chain entanglement and further changes its shear-thinning behavior, which agrees with the observed results in Figure 4.

#### 3.3.2. Dynamic Shear Behavior

**Figure 5:**
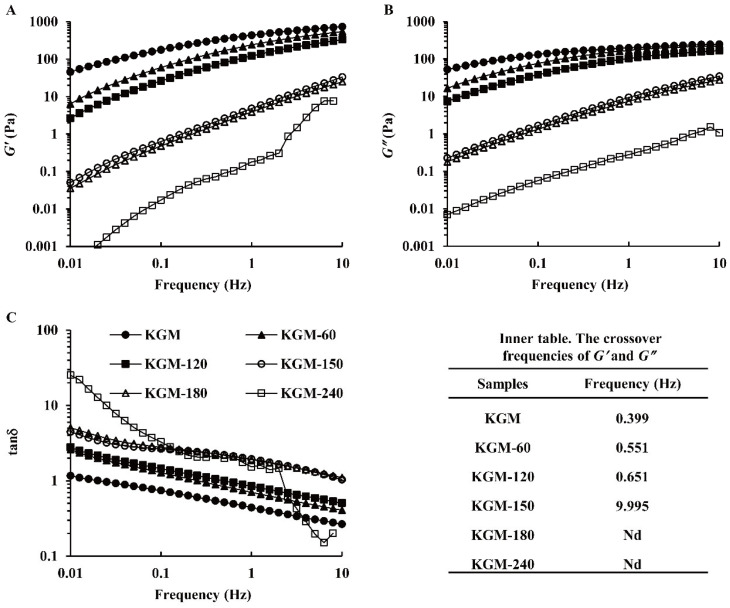
Storage modulus (G′) (**A**), loss modulus (G″) (**B**), and tanδ (**C**) of native and degraded KGMs for 60–240 min during frequency sweep.

The frequency sweep test was performed to verify the viscoelastic property and interaction of KGM molecular chains. Both the storage modulus (G′) and the loss modulus (G″) of all samples, respectively indicating elastic and viscous properties, displayed a growth with the increasing frequency (0.01–10 Hz) (Figure 5A,B), which is a typical flow property of polysaccharide solution. Generally, G″ was higher than G′, indicating a liquid-like behavior, while G″ was lower than G′, showing an approachable solid-like behavior with an elastic appearance. With the increase in frequency, the loss factor (tan δ) for all KGMs decreased markedly, as displayed in Figure 5C, which revealed the entanglement and disentanglement transformations at the molecular level [22]. KGM molecular chains disentangle during a long period of oscillation at low frequency, while these molecular chains are still entangled to form a temporary network structure during a short period of oscillation at high frequency [33]. Compared with native KGM, G′ and G″ obviously decreased and tan δ increased for degraded KGMs during 60–240 min, further revealing enzymatic hydrolysis weakened the inter- or intra-molecular interactions of KGM molecules, such as hydrogen bonds and non-covalent bonds [13,26].

As shown in Figure 5, the KGM solution had a liquid-like behavior before the crossover point, and its molecular chain started to entangle after the crossover [25]. The crossover frequencies appeared in KGM degradation for 60–150 min (Figure 5 inner table), indicating the formation of the gel network. With the increase in treatment time, the crossover point shifted to a much higher frequency, from 0.399 Hz to 0.551–9.995 Hz. The shorter chain molecules were more readily moveable to reduce the entanglement among KGM molecular chains [13], which weakened the systematic viscoelasticity and strength of the network structure [30]. This point could obviously be observed for KGM-180 and KGM-240 because their crossovers were not observed. All results illustrated that the weakened entangled network of KGM molecules during enzymatic hydrolysis and the structure of KGM were seriously damaged after a long time treatment.

#### 3.3.3. Temperature Ramp Test

**Figure 6:**
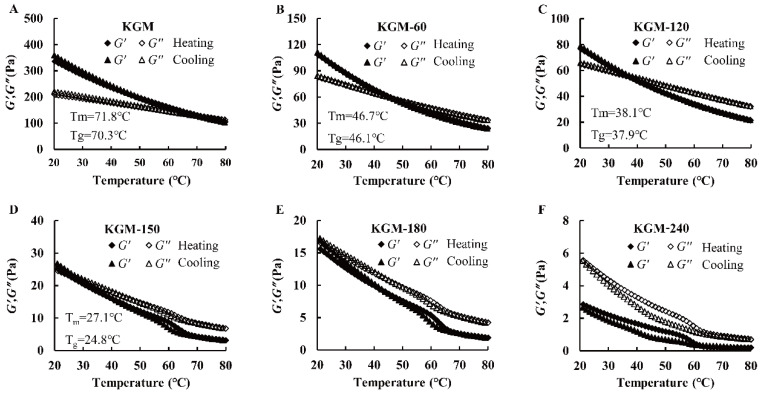
Dependence of G′ and G″ on temperature during rheological temperature ramp at a cooling and heating rate of 1 °C/min for native (**A**) and degraded KGMs for 60 min (**B**), 120 min (**C**), 150 min (**D**), 180 min (**E**) and 240 min (**F**) during frequency sweep.

The sol-gel and gel-sol transitions of all KGMs occurred at different temperatures and were monitored by an oscillation temperature sweep. The melt transition temperature (T_m_) and gelling transition temperature (T_g_) were assumed to be the crossover of dynamic modulus during heating and cooling [33]. As shown in Figure 6, the G′ and G″ decreased during the heating process from 20–80 °C, and they increased conversely in the subsequent cooling process from 80–20 °C. These tendencies were attributed to the decreased hydrogen bond of intermolecular chains and the increased molecular chain movability at high temperatures [33]. In addition, compared with native KGM with a T_m_ of 71.8 °C and a T_g_ of 70.3 °C, KGM-60, KGM-120 and KGM-150 shifted to lower levels. They were 46.7 °C and 46.1 °C, 38.1 °C and 37.9 °C, 27.1 °C and 24.8 °C, respectively (Figure 6), revealing that enzymatic treatment weakened the sol-gel and gel-sol transitions of KGM. In addition, pronounced hysteresis for KGM-180 and KGM-240 during heating-cooling cycles was found. We speculated that the different behavior of degraded KGMs with different treatment times might be related to the decline of molecular chain entanglements by thermal disruption, as well as the less thermal energy needed to disrupt the looser or weaker gel structures of KGM after enzymatic hydrolysis [24].

### 3.4. Structural Properties of Native and Degraded KGMs

#### 3.4.1. Fourier-Transform Infrared Spectroscopy (FT-IR)

**Figure 7:**
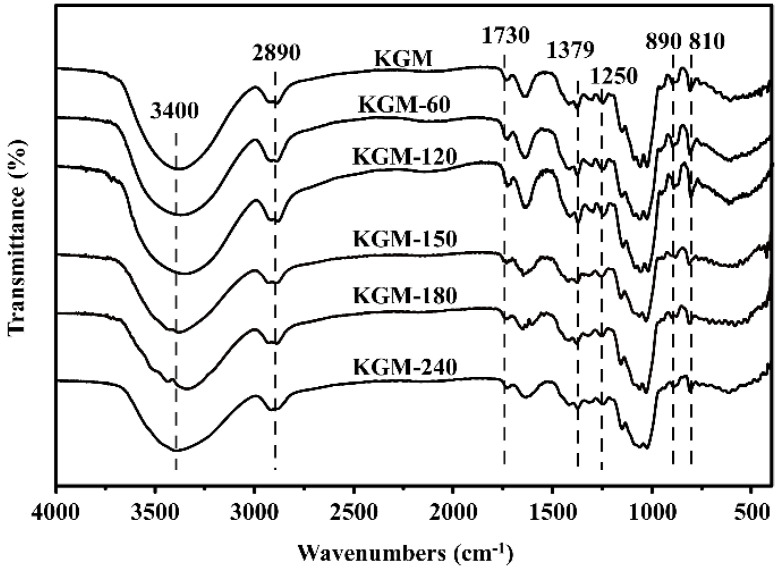
FT-IR spectra of native and degraded KGMs for 60–240 min.

The FT-IR spectra of all KGMs showed virtually identical characteristic absorption peaks from 400–4000 cm^−1^ (Figure 7). The broad absorption peak at 3400 cm^−1^ was assigned to the stretching vibration of -OH groups, and the weak peak at 2890 cm^−1^ belonged to the syntony of the C-H stretching vibration of methyl groups in the sugar ring, which are two characteristic functional groups of polysaccharides [36]. In addition, all KGMs samples had a typical absorption peak of the acetyl group at 1730 cm^−1^ associated with the stretching vibration of the C=O group [33,37], revealing that enzymatic treatment did not destroy the repeating units and primary structure of KGM. Nevertheless, the peak intensity of degraded KGMs was weaker than that of native KGM, which confirmed that the partial removal of acetyl groups might contribute to the increase in water solubility because of the hydrophobicity of acetyl groups [13].

In addition, the intense peak at around 1640 cm^−1^ is ascribed to intramolecular hydrogen bonds due to the stretching frequency of the C-O of the hydroxyl group [26,34]. Compared with native KGM, the obviously reduced intensity of degraded KGMs revealed the reduction of the free hydroxyl group after enzymatic degradation [13]. Moreover, the band at 1379 cm^−1^, assigned to the symmetric C-H bending vibration of the methyl group, as well as the absorption region around 1200 cm^−1^–1000 cm^−1^, belonged to the stretching vibrations of C-O-C or C-O-H and were also found in all KGMs, suggesting that they contain a pyranoid ring and methyl group. The observed peaks at 890 and 810 cm^−1^ belonged to the in-phase ring stretching and the deformation of the equatorial C_2_-H bond in the pyrazolyl ring of glucose and mannose, respectively [33,34]. The intensity of these peaks decreased after enzymatic treatment, which indicated that the break of chemical bonds during enzyme degradation was mainly glycosidic bond [25,30].

#### 3.4.2. Field Emission Scanning Electron Microscopy (FESEM)

**Figure 8:**
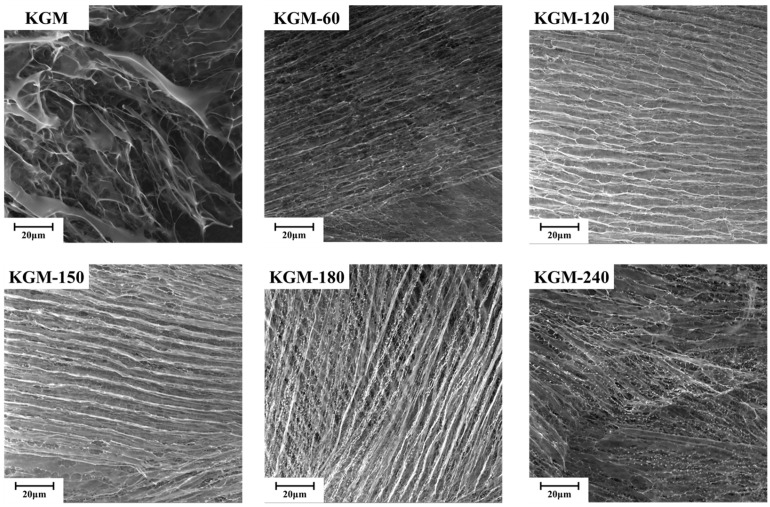
FESEM of native and degraded KGMs for 60–240 min.

As shown in Figure 8, native KGM displayed orderly and relatively compact fibrous networks and appeared like a bundle of straws, with stems well oriented in the same direction and branches randomly cross-linked. After enzyme degradation during 60–240 min, the microstructure of KGM changed notably, and its surface became porous and rough, with more irregular folds and creases along the flake, especially KGM-150, KGM-180, and KGM-240. These structural features could increase the contact areas with water, resulting in an improvement in hydration rate and capacity [19], which is in agreement with the rheological behaviors of degraded KGMs (Figure 4). The reduced chain length and increased surface wrinkles partly explain the change in rheological behaviors from the shear-thinning fluid of long polymer chains with gel networks to the Newtonian fluid of short fractions well-dispersed in water [38].

### 3.5. Partial Correlation Analysis

**Figure 9:**
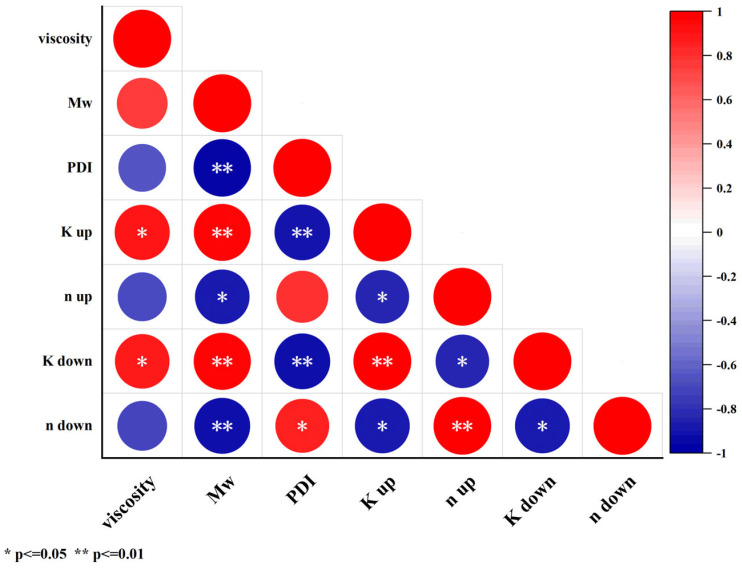
Correlation analysis of native and degraded KGMs for 60–240 min.

To further clarify the correlation between the indicators of KGM before and after enzymatic digestion, the Pearson two-tailed test was used to analyze the correlation between viscosity, molecular characteristics, and power-low parameters of KGM and DKGM. As shown in Figure 9, 8 pairs of indicators showed a highly significant correlation (*p* ≤ 0.01), and 8 pairs of indicators showed significant correlation (*p* ≤ 0.05). Among them, viscosity was a significantly correlated with K up and K down of power-low parameters; molecular characteristics were significantly correlated with power-low parameters. It can be seen that the destruction of glycosidic bonds in the molecular chain of KGM after enzymatic hydrolysis led to a decrease in molecular weight, which can significantly improve the dispersibility of KGM in water, and the decrease in the intra-chain interaction force made DKGM have a better flow.

### 3.6. Schematic Diagram of Degraded KGM by Enzymatic Hydrolysis

**Figure 10:**
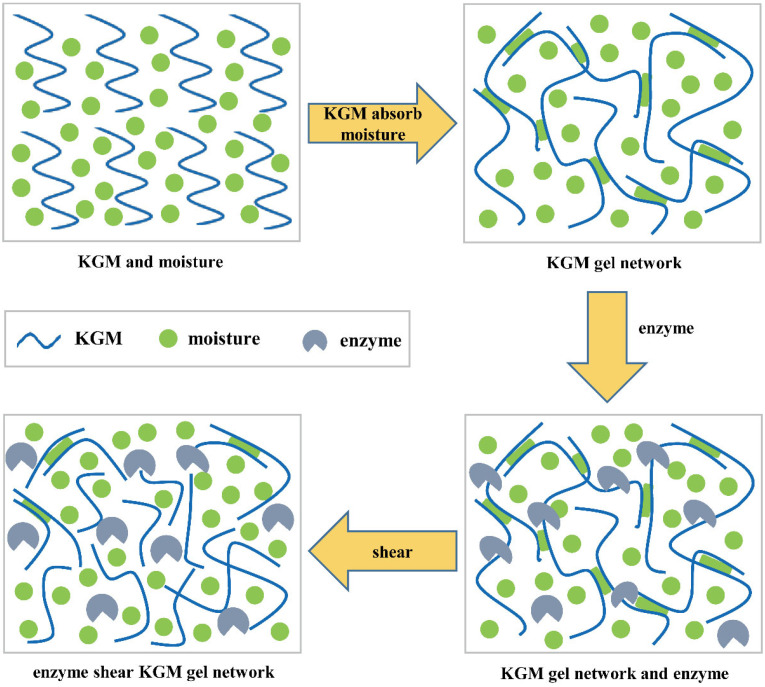
A schematic diagram describing the changes in KGM chain morphology during abalone viscera enzyme hydrolysis.

From the results above, we proved that the properties of degraded KGMs were significantly improved. A schematic model of their potential mechanisms is shown in Figure 10. KGM molecules are generally hydrated with moisture through hydrogen bonds to form gel networks [39]. However, the native KGM molecule is a long chain linked by glycosidic bonds. Thus, these molecules with high molecular weight and large granules (Figure 3 and Table 1) in solution experienced more inter- or intra-molecular interactions by entanglements and hydrogen, electrostatic, and hydrophobic bonds to form a more compact microstructure (Figure 8). The limited hydrated reaction led to the low solubility and high viscosity properties of native KGM (Figure 4, Figure 5 and Figure 6 and Table 2).

After enzymatic hydrolysis, the obtained KGM fractions had lower molecular weight and shorter chains (Figure 3 and Table 1) a result of breaking the glycoside bonds in KGM and being replaced by polar hydroxyl groups [40]. Longer enzymatic hydrolysis released more hydrophilic hydroxyl groups at the end of molecules to provide more active sites that could easily bind to water, resulting in an increase in the solubility properties of degraded KGM [41], such as KGM-60, KGM-120, and KGM-180. Moreover, the shorter chain of degraded KGM makes it easier to form a network with more irregular folds and creases along the flake (Figure 8) and increases contact areas with water, further improving the hydration of KGM [19]. Nevertheless, excessive enzymatic hydrolysis is possible to produce too short KGM fractions to form a network, such as KGM-180 and KGM-240 (Figure 3 and Figure 8 and Table 1). Hence, these degraded KGMs with damaged gel networks presented a Newtonian flow behavior similar to that of water (Figure 4, Figure 5 and Figure 6 and Table 2) [38].

## 4. Conclusions

A simple, low-cost, effective enzymatic hydrolysis was established to degrade KGM with high hydrophilicity and low viscosity properties using endo-β-1,4-glucanase from abalone viscera. The enzymatic hydrolysis of native KGM was effective in producing degraded KGM with low molecular weight and a short chain by breaking glycosidic bonds. The change of microstructure from an orderly and compact linear structure to loose and irregular structures results in more flexible chain conformation, as well as more pseudoplastic properties and better fluidity. Thus, the degraded KGM by abalone viscera enzymes with controllable molecular and rheological properties showed great potential for application in the food industry with higher additive amounts. It is interesting that after hydrolysis, the molecular weight of KGM decreases to approximately 1/4 of the native KGM, while the viscosity value decreases to 1/39 compared with the native KGM. Thus, the mechanism in the hydrolysis process of the abalone viscera enzyme toward KGM would be much more meaningful to be clarified in further research. Furthermore, the degraded KGM usually has various biological functions, such as anti-oxidation, dietary fiber, cellular protection, and prebiotic effects, that also need to be assessed in our further work.

## Figures and Tables

**Table 1:** Molecular characteristics of native and degraded KGMs for 60–240 min.

Samples	Weight Average Molecular Weight (Da)	Polydispersity Index (PDI)
KGM	1.80 × 10^6^	1.17
KGM-60	1.62 × 10^6^	1.22
KGM-120	1.24 × 10^6^	1.32
KGM-150	6.93 × 10^5^	1.76
KGM-180	5.71 × 10^5^	1.88
KGM-240	4.48 × 10^5^	1.83

**Table 2:** The Power-law parameters of native and degraded KGMs for 60–240 min.

Samples	Up Curve			Down Curve		
	K (Pa·s^n^)	n	R^2^	K (Pa·s^n^)	n	R^2^
KGM	213.004	0.174	0.910	196.709	0.186	0.929
KGM-60	131.440	0.268	0.939	128.514	0.273	0.941
KGM-120	79.287	0.343	0.970	75.699	0.361	0.976
KGM-150	17.355	0.412	0.991	16.042	0.429	0.993
KGM-180	11.259	0.417	0.996	8.518	0.483	0.995
KGM-240	1.720	0.670	0.999	1.651	0.677	0.999

## Data Availability

The data that support the findings of this study are available on request from the corresponding author, L.-C.S. The data are not publicly available due to laboratory policies and confidentiality agreements. We have fully described the experimental design, analysis, and results, as well as the process of data analysis and processing. If editors and reviewers have questions about specific data, we do our best to provide more detailed explanations and illustrations.
